# Definition of the role of chromosome 9p21 in sporadic melanoma through genetic analysis of primary tumours and their metastases

**DOI:** 10.1054/bjoc.2000.1513

**Published:** 2000-12

**Authors:** G Palmieri, A Cossu, P A Ascierto, G Botti, M Strazzullo, A Lissia, M Colombino, M Casula, C Floris, F Tanda, M Pirastu, G Castello

**Affiliations:** Institute of Molecular Genetics, C.N.R., Alghero (SS), Casella Postale, Santa Maria La Palma (Sassari), 07040, Italy; Institute of Pathology, University of Sassari, Viale San Pietro 10, Sassari, 07100, Italy; National Tumor Institute ‘G. Pascale’, Via M. Semmola, Naples, 80131, Italy; Oncologic Hospital ‘A: Businco’, A.S.L. 8, Via Jenner, Cagliari, 09100, Italy

**Keywords:** malignant melanoma, chromosome 9p21, polymerase chain reaction, microsatellite analysis, tumour progression

## Abstract

Malignant melanoma (MM) is thought to arise by sequential accumulation of genetic alterations in normal melanocytes. Previous cytogenetic and molecular studies indicated the 9p21 as the chromosomal region involved in MM pathogenesis. In addition to the CDKN genes (p16/CDKN2A, p15/CDKN2B and p19^ARF^, frequently inactivated in familial MM), widely reported data suggested the presence within this region of other melanoma susceptibility gene(s). To clearly assess the role of the 9p21 region in sporadic melanoma, we evaluated the presence of microsatellite instability (MSI) and loss of heterozygosity (LOH) in primary tumours as well as in synchronous or asynchronous metastases obtained from the same MM patients, using 9 polymorphic markers from a 17-cM region at 9p21. LOH and MSI were found in 27 (41%) and 11 (17%), respectively, out of 66 primary tumours analysed. In corresponding 58 metastases, MSI was found at higher rate (22; 38%), whereas a quite identical pattern of allelic deletions with 27 (47%) LOH+ cases were observed. Although the CDKN locus was mostly affected by LOH, an additional region of common allelic deletion corresponding to marker D9S171 was also identified. No significant statistical correlation between any 9p21 genetic alteration (LOH, MSI or both) and clinicopathological parameters was observed. © 2000 Cancer Research Campaign http://www.bjcancer.com


British Journal of Cancer (2000) 83(12), 1707–1714
© 2000 Cancer Research Campaign
doi: 10.1054/ bjoc.2000.1513, available online at http://www.idealibrary.com on IDEAL®

http://www.bjcancer.com

### Definition of the role of chromosome 9p21 in sporadic melanoma through genetic analysis of primary tumours and their metastases

**G Palmieri[1], A Cossu[2], PA Ascierto[3], G Botti[3], M Strazzullo[3], A Lissia[2], M Colombino[1], M Casula[1], C Floris[4], F Tanda[2], M Pirastu[1] and G Castello[3] for the Melanoma Cooperative Group***

[1]Institute of Molecular Genetics, C.N.R., Alghero (SS), Casella Postale, 07040 Santa Maria La Palma (Sassari), Italy; [2]Institute of Pathology, University of Sassari, Viale San Pietro 10, 07100 Sassari, Italy; [3]National Tumor Institute 'G. Pascale', Via M. Semmola, 80131 Naples, Italy; and [4]Oncologic Hospital 'A: Businco', A.S.L. 8, Via Jenner, 09100 Cagliari, Italy

**Summary** Malignant melanoma (MM) is thought to arise by sequential accumulation of genetic alterations in normal melanocytes. Previous cytogenetic and molecular studies indicated the 9p21 as the chromosomal region involved in MM pathogenesis. In addition to the CDKN genes (p16/CDKN2A, p15/CDKN2B and p19$^{ARF}$, frequently inactivated in familial MM), widely reported data suggested the presence within this region of other melanoma susceptibility gene(s). To clearly assess the role of the 9p21 region in sporadic melanoma, we evaluated the presence of microsatellite instability (MSI) and loss of heterozygosity (LOH) in primary tumours as well as in synchronous or asynchronous metastases obtained from the same MM patients, using 9 polymorphic markers from a 17-cM region at 9p21. LOH and MSI were found in 27 (41%) and 11 (17%), respectively, out of 66 primary tumours analysed. In corresponding 58 metastases, MSI was found at higher rate (22; 38%), whereas a quite identical pattern of allelic deletions with 27 (47%) LOH+ cases were observed. Although the CDKN locus was mostly affected by LOH, an additional region of common allelic deletion corresponding to marker D9S171 was also identified. No significant statistical correlation between any 9p21 genetic alteration (LOH, MSI or both) and clinicopathological parameters was observed. © 2000 Cancer Research Campaign http://www.bjcancer.com

**Keywords:** malignant melanoma; chromosome 9p21, polymerase chain reaction; microsatellite analysis; tumour progression

Malignant melanoma (MM) is usually characterized by a poor prognosis due to its high tendency to develop metastasis. Considering the small size of most primary lesions, the metastatic potential of MM is considerably greater than that of other solid tumours (Schuchter, 1997). Consequently, for MM patients the 5-year disease-free survival is progressively worsening as Clark level of invasion and Breslow thickness of primary tumours increase (falling to under 50% for patients with lesions thicker than 3 mm) (Berwick and Halpern, 1997).

Melanocytic transformation is thought to occur by sequential accumulation of genetic alterations. Although most melanomas seem to directly arise from normal melanocytes, an increasing number of evidences indicates that (i) MM progress from pre-existing melanocytic naevi and (ii) dysplastic naevi may be considered as precursors of melanoma (Haluska and Hodi, 1998).

In the pathogenesis of primary MM, silencing of tumour suppressor genes as well as activation of oncogenes have been implicated (Healy et al, 1995; Lee et al, 1997). Genetic alterations have been frequently described for human chromosomes 9p, 1p, 10q, 6q, 11q, and 18q (Peris et al, 1995; Haluska and Hodi, 1998; Healy et al, 1998). Rearrangements or deletions of the short arm of chromosome 9 seem to represent the most common genetic aberration detected in early stage MM lesions and atypical naevi, whereas loss of genetic material from other chromosomal locations might be associated with progression of melanoma (Haluska and Hodi, 1998; Birindelli et al, 2000). These findings suggest that mutation of gene(s) within chromosome 9p may occur during the initial stages of MM tumorigenesis.

Molecular and cytogenetic investigations have restricted the candidate region involved in MM pathogenesis to chromosome 9p21 (Fountain et al, 1992). A genetic locus, CDKN, with two putative tumour suppressor genes, *CDKN2A* and *CDKN2B* (also known as *p16*$^{INK4a}$ and *p15*$^{INK4b}$, respectively), which encode specific inhibitors of cyclin-dependent kinases 4 and 6 (CDK4/CDK6), has been already identified (Kamb et al, 1994a, 1994b). CDKN2A has been found inactivated by homozygous deletion or intragenic mutation at high frequency in a diverse range of tumours and tumour cell lines, including those derived from melanomas (Nobori et al, 1994; Foulkes et al, 1997; Kumar et al, 1999). Melanoma-associated mutations of CDKN2B and p19$^{ARF}$, whose transcript is derived from an alternative codon reading frame of CDKN2A, have been only detected in patients with simultaneous CDKN2A mutations (Flores et al, 1996; Liu et al, 1997).

Germline mutations of the genes from the CDKN locus have been reported in a subset of MM pedigrees. However, no

*Received 16 May 2000*
*Revised 14 August 2000*
*Accepted 16 August 2000*

*Correspondence to:* G Palmieri

*Melanoma Cooperative Group: Aprea P, Ascierto PA, Botti G, Caracò C, Castello G, Celentano E, Comella C, Daponte A, Graziano F, Mozzillo N, Parasole R, Picone A, National Tumor Institute, Naples, Italy; Bosco L, Prota G, Satriano R, University of Naples, Italy; D'Urso M, International Institute of Genetics and Biophysics, C.N.R., Naples, Italy; Palmieri G, Institute of Molecular Genetics, C.N.R., Alghero (SS), Italy disease-associated mutations of CDKN genes have been found in about half of the melanoma-prone families with evidence of linkage to 9p21 (Fitzgerald et al, 1996; Bahuau et al, 1998; Soufir et al, 1998), suggesting that additional locus/i as well as tumour suppressor gene(s) within this region may be involved in melanoma pathogenesis. In addition, the very low frequency of disease-causing alterations (by mutation or inactivation with loss of expression) of CDKN genes in sporadic MM cases with allelic losses at 9p21 is again consistent with the presence of other melanoma susceptibility gene(s) within this chromosomal area (Gonzalgo et al, 1997; Ruiz et al, 1998; Wagner et al, 1998; Kumar et al, 1999).

To further elucidate the role of the 9p21 region in sporadic melanoma and based on our large collection of MM patients (Palmieri et al, 1999), we evaluated the genetic changes associated with the metastatic progression from primary tumour to secondary lesion. Using microsatellite markers from a 17-cM region within the 9p21 chromosomal bands, we have compared allelic gains and losses (detected as microsatellite instability (MSI) and loss of heterozygosity (LOH), respectively) between primary MM tumours and synchronous or asynchronous metastases obtained from the same patients. Statistical correlations between genetic alterations and histopathology or clinical parameters were also inferred.

### MATERIALS AND METHODS

### Tissue samples and DNA extraction

Patients with histologically documented diagnosis of malignant melanoma, whose primary and metastatic tumour samples were available for genetic analysis, were selected for this study. Disease stage was recorded as IA, IB, IIA, IIB, III, or IV according to the American Joint Committee on Cancer (AJCC) guidelines. Clinical characteristics of MM patients are reported in Table 1.

A total of 66 paired samples of microdissected primary tumours and corresponding normal tissues, obtained from patients with MM, were selected for this study. Among the same patients, 58 metastatic tumour specimens (23 from synchronous and 35 from asynchronous metastases) were also available for genetic analysis. Secondary MM site has been classified as regional metastasis (R), in any regional lymph node(s) or local recurrence (in transit metastasis involving skin or subcutaneous tissue more than 2 cm from the primary tumour but not beyond the regional lymph nodes), and distant metastasis (M), in skin or subcutaneous tissue or lymph node(s) beyond the regional lymph nodes as well as any visceral metastasis. For 16 patients, we obtained tumour specimens from both regional and distant metastases (in some cases, from multiple sites). Pathological review was performed on each case to confirm the MM diagnosis on both primary and secondary lesions. Disease status at the time of diagnosis was defined depending on clinical staging as assessed by medical history, physical examination and instrumental tests.

Histopathological classification (including Breslow thickness, Clark level of invasion, and growth pattern in primary tumour) was based on the criteria of the World Health Organization. Genomic DNA was extracted from formalin-fixed paraffin-embedded samples after microdissection separating tumour tissue from adjacent stroma by light microscopy. DNA was isolated according to previously described protocols (Pisano et al, 2000). Control DNA was obtained from normal adjacent skin of the corresponding patients.

**Table 1** Patients' characteristics. Staging was according to the American Joint Committee on Cancer (AJCC) guidelines. Data refer to (**A**) time of diagnosis and (**B**) time of tumour tissue sampling

**A**

| Characteristics | Number of patients | % |
|---|---|---|
| Total analysed | 66 | |
| Males/Females | 37/29 | 56/44 |
| Median age (years) | 53 | |
| Range | 16–84 | |
| *AJCC stage* | | |
| I | 12 | 18 |
| IA/IB | 2/10 | |
| II | 31 | 47 |
| IIA/IIB | 21/10 | |
| III | 23 | 35 |
| *Primary site* | | |
| Head and neck | 8 | 12 |
| Limbs | 25 | 38 |
| Trunk | 31 | 47 |
| Unknown | 2 | 3 |

**B**

| Characteristics | Number of patients | % |
|---|---|---|
| *AJCC stage* | | |
| I + II | 4 | 6 |
| III | 48 | 73 |
| IV | 14 | 21 |
| *Site of metastasis* | | |
| Lymph node (LN) | 42 | 64 |
| LN + skin | 9 | 14 |
| LN + others | 4 | 6 |
| Skin | 5 | 7 |
| Viscerus | 2 | 3 |
| *Status* | | |
| Dead/alive | 21/40 | 32/61 |
| Lost in follow-up | 5 | 7 |

### PCR-based analysis

Primers used to amplify simple sequence repeat markers were obtained from Life Technologies (Gaithersburg, MD). 9 marker lociat 9p21 were investigated for both allelic loss and instability: D9S162, D9S974, D9S942, D9S1748, D9S171, D9S126, D9S259, D9S169 and D9S263 (telomeric to centromeric; Figure 1). All primer sequences were as reported in Genome DataBase (GDB at http://www.gdb.org). For each marker analysis, polymerase chain reaction (PCR) was performed using 25–50 ng of DNA, 0.5 μM of each specific primer, 1.5 μM $MgCl_2$, 0.2 μM dNTPs, 1 pmol of one primer end-labelled with [$\gamma$-$^{32}$P]ATP, and 1 U AmpliTaq Polymerase (PerkinElmer, Foster City, CA) under the following conditions: one cycle of enzyme activation at 94˚C for 2 min, followed by 30 cycles of denaturation at 94˚C for 30 s, primer annealing at 55–60˚C (depending on primers) for 1 min, and polymerase extension at 72˚C for 1 min. All PCR reactions were terminated with a 10-minute extension at 72˚C. All PCR reactions were performed in a 9600 Thermal cycler (PerkinElmer, Foster City, CA). Final products were diluted 1:1 in denaturing load buffer (95% formamide, 10 mM NaOH, 0.05% xylenecyanol FF, and 0.05% bromophenol blue), denatured at 94˚C for 5 min, and 4 μl were loaded for electrophoresis on 6% polyacrylamide gels containing 7.0 M urea at 80 W. The gels were dried, and exposed to Hyperfilm MP autoradiography film (Amersham) for 16 h at room temperature.

© 2000 Cancer Research Campaign

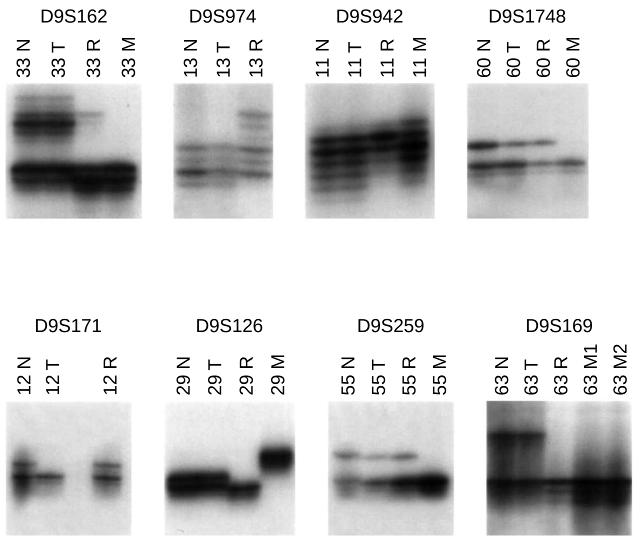

**Figure 1** Representative examples of genotyping analysis of primary melanomas and their metastases. Lanes corresponding to normal and primary tumour DNA are labelled N and T, respectively. Secondary lesions are reported as regional metastasis (R) or distant metastasis (M) (see Materials and Methods). For case 63, M1 and M2 represent distant metastases at lymph nodal and subcutaneous locations, respectively

Loss of heterozygosity (LOH) was defined by the absence or at least two-thirds reduction in the intensity of one allele in the tumour sample after comparison to the heterozygous normal tissue genotype (referred to as informative case). LOH scoring was performed by at least two investigators. Microsatellite instability (MSI) was defined by the presence of additional bands (due to deletions or insertions) in the PCR-amplified product derived from tumour DNA compared with normal DNA.

### Statistical analysis

Univariate analysis of different variables (presence of LOH and/or MSI, AJCC stage at the time of diagnosis, number and type of metastatic sites, primary tumour location, sex, age, and clinical outcome (disease-free survival and overall survival)) was performed by Pearson's Chi-Square test, using the statistical-package SPSS/7.5 per Windows.

### RESULTS

Assessment of the genetic alterations at various microsatellite loci within the chromosome 9p21 was carried out in archival tissues from 66 patients with sporadic MM. As shown in Table 1A, majority of MM patients presented localized disease (43/66 at stages I and II; 65%) at the time of diagnosis, whereas the remaining of them presented synchronous metastases in regional lymph node (23/66 at stage III; 35%). Conversely, almost all patients (62/66; 94%) presented loco-regional recurrences or distant metastases (with 21 registered deaths; 32%) at the time of this study (Table 1B); records of clinical follow-up for each patient were available, covering a median period of 78 months (range 21–172).

Paraffin-embedded tissues from primary tumours and corresponding metastases were available in 58 MM patients; therefore, in 8 cases we only obtained the primary tumour specimens. Genomic DNAs from paired normal and tumour tissues were amplified by PCR using 9 highly polymorphic markers for

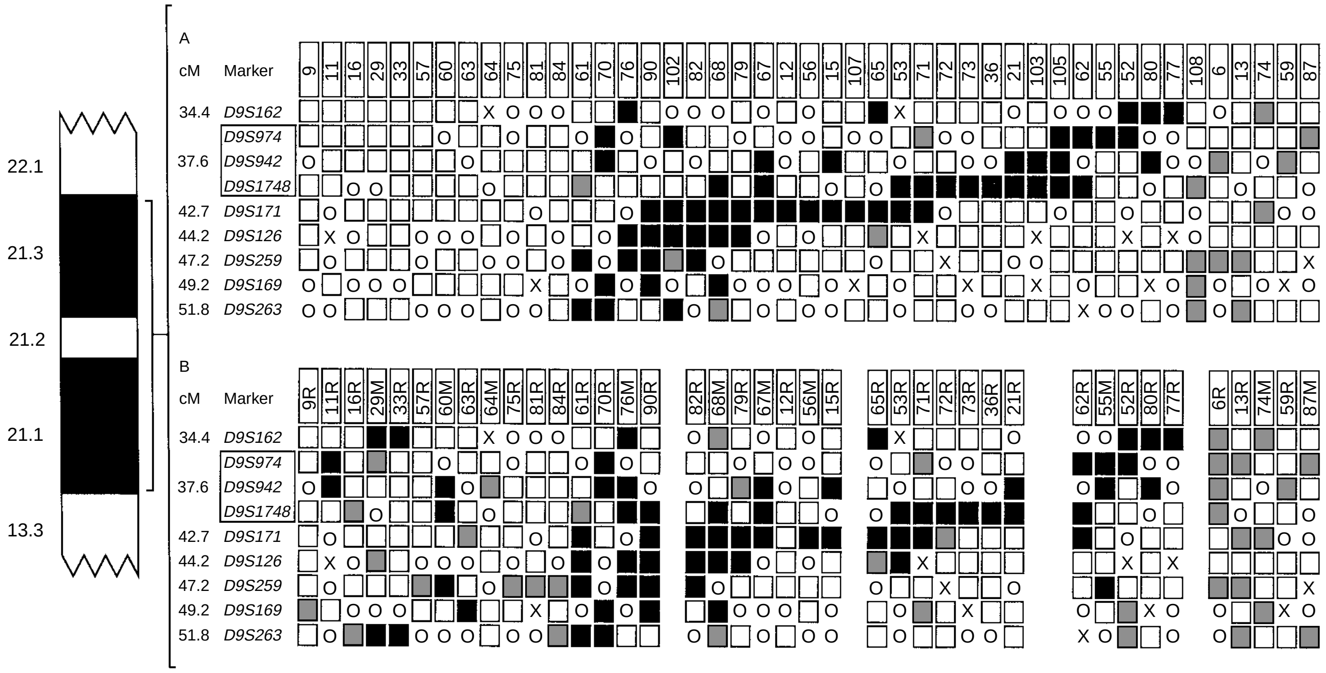

**Figure 2** Genetic alteration map at 9p21. Marker loci are ordered telomeric to centromeric. Genetic position of each marker is indicated in centiMorgans (cM), as reported in Marshmed map (at: http://www.marshmed.org). Markers spanning the CDKN locus are boxed. Patients are indicated by their number in the series. Black boxes indicate LOH. Empty white boxes indicate retained heterozygosity. White boxes with **O** or **X** indicate constitutional homozygosity or no data, respectively. Grey boxes indicate microsatellite instability. Patterns of genetic alteration among (**A**) primary melanomas, and (**B**) secondary tumours (R and M, regional and distant metastasis, respectively)

microsatellite loci spanning about 17-cM at 9p21: cen-D9S263-D9S169- D9S259- D9S126- D9S171- D9S1748- D9S942-D9S974- D9S162-tel. The microsatellite markers D9S1748, D9S942 and D9S974 are physically positioned within a 50-kb region spanning the CDKN locus (including the genes p16/CDKN2A and p15/CDKN2B). Changes in electrophoretic mobility of the microsatellite markers in tumours, after comparison with corresponding normal DNAs, permitted to define the genetic profile of primary and metastatic MM lesions at 9p21. In Figure 1, typical examples of such changes (referred to as loss of heterozygosity, LOH, and microsatellite instability, MSI) are presented for cases in which substantially different patterns of genetic alteration were observed between primary tumours and their metastases.

In cases 33 and 63 of Figure 1 (with markers D9S162 and D9S169, respectively), a complete deletion of one allele was observed in regional or distant metastases (R or M, respectively; see Materials and Methods for relative classification) but not in primary tumours (T), whereas in other two cases, 60 and 55 (with markers D9S162 and D9S169, respectively), LOH was only present in distant recurrences. Detection of additional bands, indicating the presence of MSI, in regional metastasis of case 13 (with marker D9S974) as well as particularly evident rearrangements at loci D9S942 and D9S126 in regional or distant metastases of cases 11 and 29, respectively (in both cases, allelic loss in R samples and MSI in M tissues were observed; on this regard, an insertion of dinucleotide repeats in the retained allele of 29R sample could explain the presence of a completely new allele in 29 M sample) are also reported in Figure 1. To avoid any false positive or negative result due to PCR-based artifacts, all unclear or ambiguous data were confirmed in replicated experiments (in some cases, also using different electrophoretic conditions to better separate the PCR products).

Overall, analyses of multiple metastatic tumours from the same patients revealed only a small fraction of cases with LOH detected exclusively in the secondary MM lesions. In fact, fine deletion mapping of the 9p21 region using the 9 microsatellite markers showed minimal differences in patterns of loss and retention between primary melanomas and their metastases. In Figure 2, all primary and secondary MM cases with at least one marker carrying a genetic alteration (LOH or MSI) at 9p21 are reported. Presence of allelic deletions was revealed in 27 out of 66 primary tumours (41%), whereas MSI was found in 11/66 (17%) specimens at the same level (Figure 2A). In metastatic tumour samples, while marker instability (associated or not to allelic losses) became more prevalent (22/58, 38%), a quite identical pattern of allelic deletions was observed, except for a slight increase of the total number of LOH+ cases or the presence of larger deletions in some regions already affected in primary MM tumours (Figure 2B). In particular, microsatellite analysis of all secondary lesions from the 36 patients negative for LOH in primary melanomas revealed 5 new LOH+ cases (11R, 29M, 33R, 60M, and 63R), bringing the total to 27/58 (47%) secondary tumours with allelic deletion in at least one locus (Figure 2B). Altogether, genetic alterations at 9p21 (including LOH and/or MSI) were found in 33/66 (50%) primary MM tumours and 39/58 (67%) corresponding metastatic lesions.

**Table 2** Results of microsatellite analysis at 9p21 in primary and secondary MM lesions. Patients are grouped according to the type of tumour samples analysed. 9p21+ indicates the presence of 9p21 genetic alterations in at least one sample (from primary and/or secondary lesions)

| Sample (patients) | 9p21+ | Tumour | | R / M | |
|---|---|---|---|---|---|
| | | MSI+ | LOH+ | MSI+ | LOH+ |
| T only (8) | 5 | 2 | 4 | – | – |
| T + R (37) | 23 | 4 | 15 | 12 | 14 |
| T + M (4) | 3 | 0 | 2 | 1 | 2 |
| T + R + M (17) | 14 | 5 | 6 | 9 | 11 |
| TOTAL (66) | 45 | 11 | 27 | 22 | 27 |

T, primary tumour; R, regional metastasis; M, distant metastasis (see Materials and Methods).

As summarized in Table 2, two thirds (45/66; 68%) of MM patients presented 9p21 genetic alterations in at least one sample (from primary and/or secondary lesions). With the exclusion of the 8 MM cases for whom only tumour samples were available, for the majority (41/58; 71%) of patients microsatellite analysis was performed at both primary melanoma and one metastatic site (regional or distant metastasis) (Table 2). In the remaining 17 cases, multiple metastases from the same patients were analysed (Table 2), and the 14 MM cases found positive for genetic alterations at 9p21 are shown in Figure 3.

The observed frequencies of allelic gains and losses for the nine markers at 9p21 used in this study are reported in Table 3. LOH

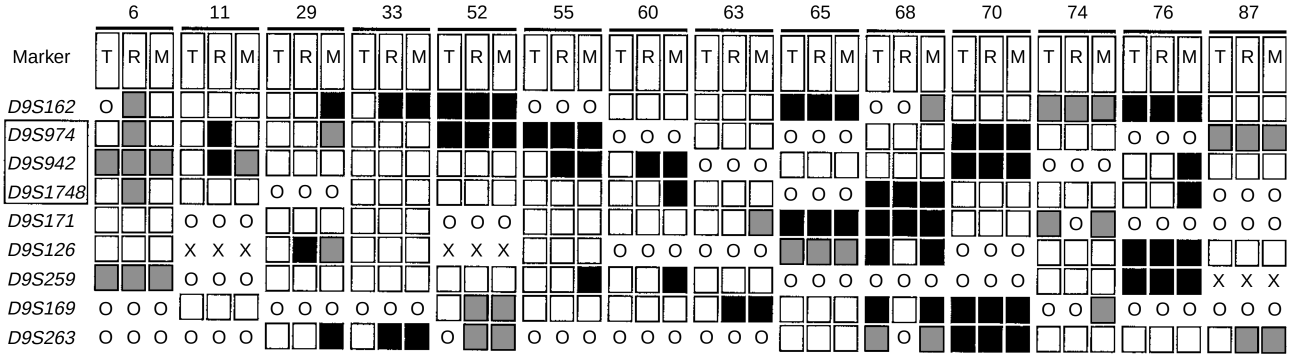

**Figure 3** Results of microsatellite analysis at 9p21 among the 14 patients with multiple metastases. T, primary tumour; R, regional metastasis; M, distant metastasis (see Materials and Methods). Markers spanning the CDKN locus are boxed. Meaning of boxes and symbols is as in legend of Figure 2

*© 2000 Cancer Research Campaign*

ranged from 9% for both D9S259 and D9S263 to 27% for D9S171, in primary tumours, and from 12% for D9S974 to 28% for D9S1748 in secondary MM lesions, with an average of 66% informativeness at each locus (Table 3A). MSI was detected at very low levels in primary melanomas (ranging from 1.5% for D9S171 to 6.3% for D9S259), with an increased incidence in secondary tumours (ranging from 4% for D9S126 to 12% for D9S263) (Table 3B).

The 27 primary tumours with LOH were separately reported in Figure 4. Most melanomas (20, 74%) presented the involvement of the markers (D9S1748, D9S942, and D9S974) spanning the CDKN locus (Fig. 4A). In this group, four cases (70, 76, 102 and 65), showing heterozygosity at loci surrounding the CDKN genes as well as LOH in the telomeric and centromeric regions bracketing this locus (see Fig. 4A), could represent tumours carrying large allelic deletions (interspersed heterozygosity has been described as probably due to the presence of contaminating stromal cells (Cairns et al, 1995)).

For 5 of these 20 patients (cases 67, 70, 80, 103 and 105; Figure 4A), peripheral blood samples were available and additional

**Table 3** Frequencies of LOH and MSI for each 9p21 locus marker in primary and secondary MM lesions. Loci are listed telomeric to centromeric, and the three markers from the CDKN locus are boxed. (A) LOH frequency is calculated taking into account the number of the informative samples for each marker. (B) MSI frequency is determined on the basis of the analysed cases

**A**

| | Primary tumours | | | Secondary lesions | | |
|---|---|---|---|---|---|---|
| Marker | Informative/ Analysed cases | LOH+ cases | LOH % | Informative/ Analysed cases | LOH+ cases | LOH % |
| D9S162 | 42/64 | 5 | 12 | 38/56 | 7 | 18 |
| D9S974 | 47/66 | 6 | 13 | 41/58 | 5 | 12 |
| D9S942 | 46/66 | 7 | 15 | 40/58 | 9 | 23 |
| D9S1748 | 48/66 | 11 | 23 | 43/58 | 12 | 28 |
| D9S171 | 49/66 | 13 | 27 | 44/58 | 12 | 27 |
| D9S126 | 39/57 | 6 | 15 | 36/53 | 7 | 19 |
| D9S259 | 44/64 | 4 | 9 | 39/56 | 6 | 15 |
| D9S169 | 31/57 | 3 | 10 | 28/53 | 4 | 14 |
| D9S263 | 32/60 | 3 | 9 | 29/52 | 4 | 14 |

**B**

| | Primary tumours | | | Secondary lesions | | |
|---|---|---|---|---|---|---|
| Marker | Analysed cases | MSI+ cases | MSI % | Analysed cases | MSI+ cases | MSI % |
| D9S162 | 64 | 1 | 1.6 | 56 | 3 | 5 |
| D9S974 | 66 | 2 | 3.0 | 58 | 5 | 9 |
| D9S942 | 66 | 2 | 3.0 | 58 | 4 | 7 |
| D9S1748 | 66 | 2 | 3.0 | 58 | 3 | 5 |
| D9S171 | 66 | 1 | 1.5 | 58 | 4 | 7 |
| D9S126 | 57 | 1 | 1.8 | 53 | 2 | 4 |
| D9S259 | 64 | 4 | 6.3 | 56 | 6 | 11 |
| D9S169 | 57 | 1 | 1.8 | 53 | 4 | 8 |
| D9S263 | 60 | 3 | 4.9 | 52 | 6 | 12 |

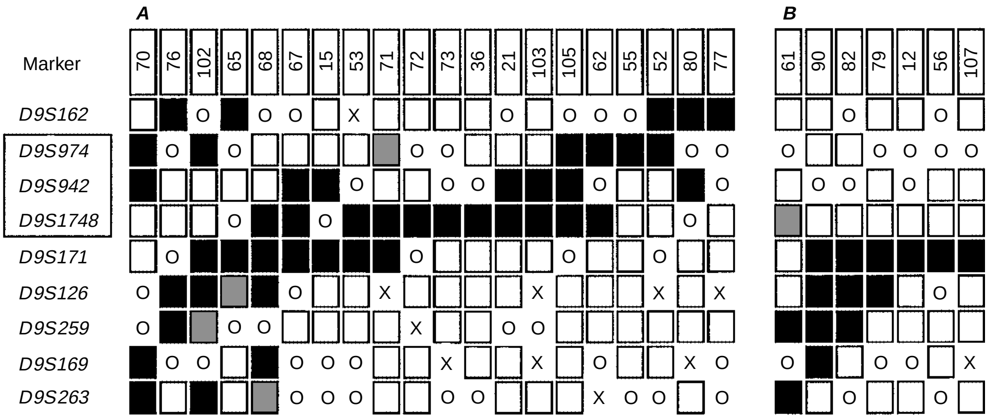

**Figure 4** Classification of the 27 LOH+ primary tumours. Patients were grouped according to the presence of allelic deletions involving (A) or not (B) the markers (boxed) from the CDKN locus. Meaning of boxes and symbols is as in legend of Figure 2

specimens from microdissected primary tumours were obtained. In these cases, direct sequencing of the corresponding genomic DNAs was performed for exons 1, 2, and 3 of p16/CDKN2A. Although LOHs were particularly evident within the region of the CDKN locus for all 5 primary melanomas analysed (see Figure 4A), no somatic or germline mutations were detected in any of these exons (data not shown).

Among the remaining 7 (26%) LOH+ primary melanomas, allelic deletions were found confined to regions centromeric to CDKN locus (Figure 4B). Interestingly, LOH analysis in this subset of patients seem to narrow the region of allelic loss to D9S171, which remained the only deleted marker in three primary tumours (cases 12, 56 and 107; Figure 4B). Altogether, LOHs at the locus D9S171 were detected in 13/27 (48%) primary MM tumours.

No significant statistical correlations between the presence of 9p21 genetic alterations (LOH and/or MSI) and any histological or clinical parameter (Breslow, Clark, primary site, number and type of metastatic sites, sex, age, and clinical outcome) was observed. After classification by disease stage (following the AJCC guidelines), a quite identical distribution was observed among the groups of MM patients negative (6/12 (50%) stage I, 15/31 (48%) stage II, and 11/23 (48%) stage III patients) or positive (6/12 (50%) stage I, 16/31 (52%) stage II, and 12/23 (52%) stage III patients) for genetic alterations at 9p21. With the exclusion of the 5 MM patients lost during the follow-up (see Table 1B), no significant differences in disease-free survival (DFS) or overall survival (OS) rates were observed between the two groups, without (31 patients: median DFS, 21 months; median OS, 43 months) and with (30 patients: median DFS, 22 months; median OS, 40 months) 9p21 genetic alterations.

### DISCUSSION

In this study we systematically investigated the genetic profile at chromosome 9p21 of primary tumours and corresponding metastases in a number of patients with malignant melanoma. Among 66 primary MM lesions, screening with 9 markers covering a 17-cM distance at this chromosomal location allowed to identify a large subset (27; 41%) of tumours carrying at least one allelic deletion. LOH at one or more polymorphic microsatellite markers on 9p21 has been reported ranging from 20% to 71% in uncultured sporadic primary melanomas, whereas 63% to 96% of the MM cell lines have been demonstrated to harbour allelic losses within the same region (Gonzalgo et al, 1997; Haluska and Hodi, 1998; Kumar et al, 1999). In our study, detection of 9p21 allelic losses at quite same rate in primary tumours and corresponding metastases (41% vs 47%, respectively) and, mostly, the presence of highly similar patterns of LOH among these lesions suggested that such genetic alterations may occur before tumour dissemination, playing a role in the development of melanoma. These findings are consistent with the view that LOH at 9p21 is an early event in the pathway of progression to melanoma (Fountain et al, 1992; Healy et al, 1995; Morita et al, 1998). Nonetheless, the frequency of allelic loss here reported was lower than that usually found in MM cell lines, confirming previous data and thus emphasizing the importance of examining uncultured tumours in order to define all events involved in tumorigenesis and tumour progression (Ruben et al, 2000).

In our series, presence of at least one unstable microsatellite marker on 9p21 identified a minority of primary MM tumours (11/66; 17%) with MSI, which may reflect a defect in genes involved in DNA replication fidelity. Slightly higher frequencies (ranging from 20% to 26%) have been reported by some authors, not only in primary melanomas but also in other melanocytic lesions (including dysplastic and/or common nevi) (Talwalkar et al, 1998; Birindelli et al, 2000; Ruben et al, 2000). When the same series of MM patients has been analysed for the presence of microsatellite instability in the corresponding metastases, we found a higher incidence (22/58, 38%) of this genetic alteration, indicating that MSI may probably represent an alternative pathway of tumour cell evolution in malignant melanoma.

Overall, comparison of the data on MSI within the 9p21 region during melanoma progression, as inferred by PCR-based analysis on either uncultured primary tumours and their metastases or cell lineages from MM tissues (the latter, characterized by abundant microsatellite instability (Talwalkar et al, 1998; Birindelli et al, 2000)), strongly suggests the existence of pathogenetic mechanisms progressively inducing rearrangements of this chromosomal area in order to provide a selective advantage during malignant evolution. In other words, since tumorigenesis and tumour progression can be considered an evolutionary process, one could hypothesize that genetic instability at 9p21 may contribute to selective pressures during melanoma progression.

Deletion mapping of 9p21 revealed that at least two discrete regions of allelic loss were present in primary melanomas: the first, spanning the CDKN locus and including the markers D9S974, D9S942, and D9S1748 (located in a 50-kb DNA fragment comprising the exons of the genes p16/CDKN2A, p15/CDKN2B and $p19^{ARF}$), showed the highest rate of allelic deletions (20/27; 74%); the second, including the marker D9S171 (centromeric to the CDKN locus), was found involved in about half (13/27; 48%) LOH+ primary tumours, representing the region of common allelic loss in the 7 (26%) remaining primary melanomas with LOH not associated to the CDKN locus (see Figure 4).

Following the isolation of p16/CDKN2A and p15/CDKN2B, several studies on a large number of cell lines and primary tumours have been published, suggesting that this locus may be one of the most frequently altered in human cancer (Haluska and Hodi, 1998). Studies on melanoma have clearly indicated that *p16/CDKN2A* gene is altered in the vast majority of melanoma cell lines (homozygous deletion rate is roughly 60%; an additional 15% to 20% of lines presents point mutations in the gene, for a total mutation rate of approximately 75% to 80%) (Flores et al, 1996; Pollock et al, 1996; Foulkes et al, 1997). Conversely, the rate of p16/CDKN2A alteration is much lower in sporadic uncultured melanomas, ranging from less than 10% in primary tumours to 20–25% in secondary lesions (Reed et al, 1995; Ruiz et al, 1998; Fujimoto et al, 1999). This low mutation rate in contrast to a markedly higher incidence of hemizygous deletions reported for 9p21 in sporadic primary melanomas (20–71% including our results of 41%; see above) strongly address to search additional putative tumour suppressor gene(s) within the genomic DNA regions surrounding the CDKN locus.

Although presence of a second region of allelic loss that does not include the CDKN locus has been argued (Merbs and Sidransky, 1999), several PCR-based LOH studies on melanoma have indicated some loci lying centromeric or telomeric to the CDKN genes within the 9p21-p22 chromosomal area (Peris et al, 1995; Ohta et al, 1996; Kumar et al, 1999). The region of allelic loss spanning the D9S171 locus here identified is consistent with

 © 2000 Cancer Research Campaign that described by Ohta et al (1996), who found the D9S171 locus deleted in 47% of informative primary melanomas. However, this region should be further analysed by increasing the marker density in order to define the minimal genomic segment to be subsequently investigated for the presence of candidate gene(s). To date, additional polymorphic markers across the D9S1748-D9S126 interval are not available in the Genome Database, and thus they must be developed starting from sequenced DNA (which is now being reported in public databases). Interestingly, a high frequency of LOH at 9p21, including the D9S171 locus, has been described for other types of cancer (Ohgaki et al, 1999; Perinchery et al, 1999), again confirming that this region may harbour several tumour suppressor genes involved in carcinogenesis.

Absence of germline or somatic mutations of p16/CDKN2A in 5 MM patients with hemizygous deletions affecting the CDKN locus could not exclude the presence of intragenic mutations for the other two genes from the same locus (though p15/CDKN2B and $p19^{ARF}$ have been usually found mutated in patients with simultaneous p16/CDKN2A mutations (Flores et al, 1996; Liu et al, 1997)) or of alternative mechanisms inactivating these genes on the retained alleles at somatic level. Since a 5′ CpG island upstream of p16/CDKN2A has been found methylated in several primary tumours and cell lines (Little and Wainwright, 1995; Merlo et al, 1995; Costello et al, 1996; Batova et al, 1997), examination of the methylation status for this gene should be included in the analysis of uncultured primary and metastatic tumours. However, data on this regard are lacking also for the difficulty in setting adequate protocols for the analysis of small amounts of tumour cells from microdissected material. Nonetheless, melanoma-associated mutations within the promoter regions of the CDKN genes may also affect their expression by creation of aberrant initiation codons, as already demonstrated for p16/CDKN2A (Liu et al, 1999).

Altogether, our results on a limited number of MM cases and, mainly, the findings widely reported suggest that in sporadic melanoma the inactivation by deletion or mutation or gene silencing of p16/CDKN2A might be selected during the tumour progression or, especially, during the establishment and propagation of MM cells in culture (Healy et al, 1996; Ruben et al, 2000). As also recently reported (Fujimoto et al, 1999), inactivation of p16/CDKN2A seems thus not to be as frequent in primary melanomas as in cell lines. Conversely, in familial melanoma evidences strongly implicate p16/CDKN2A mutations in determining predisposition to the disease (Borg et al, 1996; Smith-Sorenson and Hoving, 1996; Zuo et al, 1996; Platz et al, 1997; Soufir et al, 1998).

An additional feature in our series was the presence of few MM patients (6/58, 10%) showing a discontinuous pattern of genetic alteration at 9p21 during tumour progression (cases 6, 11, 12, 29, 68, and 74; see Figures 2 and 3). For example, the case 12 at locus D9S171 (Figure 1) showed LOH in primary tumour and retention of heterozygosity (thus, no alteration) in its regional metastasis. Therefore, it appears that in this case at least two independent subclones have been generated: one lost material at 9p21 and grew within the primary lesion, whereas the other retained both alleles but metastasized to regional lymph node. Although these results have been demonstrated only for a subset of our MM patients, this could be an indication that in sporadic melanoma (and probably in other human cancers) the source of tumour cell heterogeneity may reside in the cell population at the early stage of tumorigenesis. One could speculate that such heterogeneous population could evolve independently in the primary melanoma and its metastases, thus suggesting that a linear progression model may not always account for the progression of primary tumours to metastases.

Finally, no genetic alteration at 9p21 (LOH or MSI or both) was significantly correlated with clinicopathological features (such as AJCC stage of disease, DFS, or OS) of MM patients in our series. Univariate analysis only showed a significant association between disease stage and clinical outcome (particularly for overall survival, with a median OS of 67 months in stage I, 42 months in stage II, and 31 months in stage III patients; $P = 0.003$), confirming the predictive value of clinical stage as prognostic factor. Therefore, further studies are needed to identify genetic alterations in primary melanomas which could have clinical relevance as tumour progression markers.

### AKNOWLEDGEMENTS

Authors like to thank Dr Assunta Criscuolo, for data management, and Dr Egidio Celentano, for statistical analysis. Work was supported by Ricerca Finalizzata Ministero della Sanità (FSN), Regione Autonoma della Sardegna (Assessorato alla Programmazione), and CNR 'Target Project on Biotechnology'.

### REFERENCES

Bahuau M, Vidaud D, Jenkins RB, Bieche I, Kimmel DW, Assouline B, Smith JS, Alderete B, Cayuela J-M, Harpey J-P, Caille B and Vidaud M (1998) Germ-line deletion involving the INK4 locus in familial proneness to melanoma and nervous system tumors. *Cancer Res* **58**: 2298–2303

Batova A, Diccianni MB, Yu JC, Nobori T, Link MP, Pullen J and Yu AL (1997) Frequent and selective methylation of p15 and deletion of both p15 and p16 in T-cell acute lymphoblastic leukemia. *Cancer Res* **57**: 832–836

Berwick M and Halpern AH (1997) Melanoma epidemiology. *Curr Opin Oncol* **9**: 178–182

Birindelli S, Tragni G, Bartoli C, Ranzani GN, Rilke F, Pierotti MA and Pilotti S (2000) Detection of microsatellite alterations in the spectrum of melanocytic nevi in patients with or without individual or family history of melanoma. *Int J Cancer* **86**: 255–261

Borg A, Johansson U, Johansson O, Håkansson S, Westerdahl J, Måsbäck A, Olsson H and Ingvar C (1996) Novel p16 mutation in familial melanoma in Southern Sweden. *Cancer Res* **56**: 2497–2500

Cairns P, Polascik TJ, Eby Y, Tokino K, Califano J, Merlo A, Mao L, Herath J, Jenkins R, Westra W et al. (1995) Frequency of homozygous deletion at p16/CDKN2 in primary human tumours. *Nat Genet* **11**: 210–212

Costello JF, Berger MS, Huang HS and Cavenee WK (1996) Silencing of p16/CDKN2 expression in human gliomas by methylation and chromatin condensation. *Cancer Res* **56**: 2405–2410

Fitzgerald MG, Harkin DP, Silva-Arrieta S, MacDonald DJ, Lucchina LC, Unsal H, O'Neil E, Koh J, Finkelstein DM, Isselbacher KJ, Sober AJ and Haber DA (1996) Prelevance of germ-line mutations in p16, $p19^{ARF}$, and CDK4 in familial melanoma: analysis of a clinic-based population. *Proc Natl Acad Sci USA* **93**: 8541–8545

Flores JF, Walker GJ, Glendening JM, Haluska FG, Castresana JS, Rubio MP, Pastorfide GC, Boyer LA, Kao WH, Bulyk ML, Barnhill RL, Hayward NK, Housman DE and Fountain JW (1996) Loss of the $p16^{INK4a}$ and $p15^{INK4b}$ genes, as well as neighboring 9p21 markers, in sporadic melanoma. *Cancer Res* **56**: 5023–5032

Foulkes WD, Flanders TY, Pollock PM, and Hayward NK (1997) The CDKN2A (p16) gene and human cancer. *Molec Med* **3**: 5–20

Fountain JW, Karayiorgou M, Ernstoff MS, Kirkwood JM, Vlock DR, Titus-Ernstoff L, Bouchard B, Vijayasaradhi S, Houghton AN, Lahti J, Kidd VJ, Housman DE and Dracopoli NC (1992) Homozygous deletion within human chromosome band 9p21 in melanoma. *Proc Natl Acad Sci USA* **89**: 10557–10561

Fujimoto A, Morita R, Hatta N, Takehara K and Takata M (1999) p16INK4a inactivation is not frequent in uncultured sporadic primary cutaneous melanoma. *Oncogene* **18**: 2527–2532

© 2000 Cancer Research Campaign

Gonzalgo ML, Bender CM, You EH, Glendening JM, Flores JF, Walker GJ, Hayward NK, Jones PA and Fountain JW (1997) Low frequency of p16/CDKN2A methylation in sporadic melanoma: comparative approaches for methylation analysis of primary tumors. *Cancer Res* **57**: 5336–5347

Haluska FG and Hodi FS (1998) Molecular genetics of familial cutaneous melanoma. *J Clin Oncol* **16**: 670–682

Healy E, Rehman I, Angus B and Rees JL (1995) Loss of heterozygosity in sporadic primary cutaneous melanoma. *Genes Chromosomes Cancer* **12**: 152–156

Healy E, Sikkin S and Rees JL (1996) Infrequent mutation of p16INK4 in sporadic melanoma. *J Invest Dermatol* **107**: 318–321

Healy E, Belgaid C, Takata M, Harrison D, Zhu NW, Burd DA, Rigby HS, Matthews JN and Rees JL (1998) Prognostic significance of allelic losses in primary melanoma. *Oncogene* **16**: 2213–2218

Kamb A, Shattuck-Eidens D, Eeles R, Liu Q, Gruis NA, Ding W, Hussey C, Tran T, Miki Y, Weaver-Feldhaus J, McClure M, Aitken JF, Anderson DE, Bergman W, Frants R, Goldar DE, Green A, MacLennan R, Martin NG, Meyer LJ, Youl P, Zone JJ, Skolnick MH and Cannon Albright LA (1994a) Analysis of the p16 gene (CDKN2) as a candidate for the chromosome 9p melanoma susceptibility locus. *Nature Genet* **8**: 22–26

Kamb A, Gruis NA, Weaver-Feldhaus J, Liu Q, Harshman K, Tavtigian SV, Stockert E, Day RS, Johnson BE and Skolnick MH (1994b) A cell cycle regulator potentially involved in genesis of many tumor types. *Science* **264**: 436–440

Kumar R, Smeds J, Lundh-Rozell B and Hemminki K (1999) Loss of heterozygosity at chromosome 9p21 (INK4-p14ARF locus): homozygous deletions and mutations in the p16 and p14ARF genes in sporadic primary melanomas. *Melanoma Res* **9**: 138–147

Lee JY, Dong SM, Shin MS, Kim SY, Lee SH, Kang SJ, Lee JD, Kim CS, Kim SH and Yoo NJ (1997) Genetic alterations of p16INK4a and p53 genes in sporadic dysplastic nevus. *Biochem Biophys Res Commun* **237**: 667–672

Little M and Wainwright B (1995) Methylation and p16: suppressing the suppressor. *Nature Med* **1**: 633–634

Liu L, Godstein AM, Tucker MA, Brill H, Gruis NA, Hogg D and Lassam NJ (1997) Affected members of melanoma-prone families with linkage to 9p21 but laking mutations in CDKN2A do not harbor mutations in the coding regions of either CDKN2B or $p19^{ARF}$. *Genes Chromosomes Cancer* **19**: 52–54

Liu L, Dilworth D, Gao L, Monzon J, Summers A, Lassam N and Hogg D (1999) Mutation of the CDKN2A 5′ UTR creates an aberrant initiation codon and predisposes to melanoma. *Nat Genet* **21**: 128–132

Merbs SL and Sidransky D (1999) Analysis of p16 (CDKN2/MTS-1/INK4A) alterations in primary sporadic uveal melanoma. *Invest Ophthalmol Vis Sci* **40**: 779–783

Merlo A, Herman JG, Mao L, Lee DJ, Gabrielson E, Burger PC, Baylin SB and Sidransky D (1995) 5′ CpG island methylation is associated with transcriptional silencing of the tumor suppressor p16/CDKN2/MTS1 in human cancers. *Nature Med* **1**: 686–692

Morita R, Fujimoto A, Hatta N, Takehara K and Takata M (1998) Comparison of genetic profiles between primary melanomas and their metastases reveals genetic alterations and clonal evolution during progression. *J Invest Dermatol* **111**: 919–924

Nobori T, Miura K, Wu DJ, Lois A, Takabayashi K and Carson DA (1994) Deletions of the cyclin dependent Kinase-4 inhibitor gene in multiple human cancers. *Nature* **368**: 753–756

Ohgaki K, Minobe K, Kurose K, Iida A, Habuchi T, Ogawa O, Kubota Y, Akimoto M and Emi M (1999) Two target regions of allelic loss on chromosome 9 in urinary-bladder cancer. *Jpn J Cancer Res* **90**: 957–964

Ohta M, Berd D, Shimizu M, Nagai H, Cotticelli MG, Mastrangelo M, Shields JA, Shields CL, Croce CM and Huebner K (1996) Deletion mapping of chromosome 9p21-p22 surronding the CDKN2 locus in melanoma. *Int J Cancer* **65**: 762–767

Palmieri G, Strazzullo M, Ascierto PA, Satriano SM, Daponte A and Castello G for the Melanoma Cooperative Group (1999) Polymerase chain reaction-based detection of circulating melanoma cells as an effective marker of tumor progression. *J Clin Oncol* **17**: 304–311

Perinchery G, Bukurov N, Nakajima K, Chang J, Li LC and Dahiya R (1999) High frequency of deletion on chromosome 9p21 may harbor several tumor-suppressor genes in human prostate cancer. *Int J Cancer* **83**: 610–614

Peris K, Keller G, Chimenti S, Amantea A, Kerl H and Hofler H (1995) Microsatellite instability and loss of heterozygosity in melanoma. *J Invest Dermatol* **105**: 625–628

Pisano M, Cossu A, Persico I, Palmieri G, Angius A, Casu G, Palomba G, Sarobba MG, Rocca PC, Dedola MF, Olmeo N, Pasca A, Budroni M, Marras V, Pisano A, Farris A, Massarelli G, Pirastu M and Tanda F (2000) Identification of a founder BRCA2 mutation in Sardinia. *Br J Cancer* **82**: 553–559

Platz A, Hanson J, Mansson-Brahme E, Lagerlöf B, Linder S, Lundqvist E, Sevigny P, Inganäs M and Ringborg U. (1997) Screening of germline mutations in the CDKN2A and CDKN2B genes in Swedish families withh ereditary cutaneous melanoma. *J Natl Cancer Inst* **89**: 697–702

Pollock PM, Pearson JV and Hayward NK (1996) Compilation of somatic mutations of the CDKN2 gen in human cancers: non-random distribution of base substitutions. *Genes Chromosom Cancer* **15**: 77–88

Reed JA, Loganzo F, Shea CR, Walker GJ, Flore JF, Glendening JM, Bogdany JK, Shiel MJ, Haluska FG, Fountain JW and Albino AP (1995) Loss of expression of the p16/cyclin-dependent kinase inhibitor 2 tumor suppressor gene in melanocityc lesion correlates with invasive stage of tumor progression. *Cancer Res* **55**: 2713–2718

Ruben A, Babilas P, Baron JM, Hofheinz A, Neis M, Sels F and Sporkert M (2000) Analysis of tumor cell evolution in a melanoma: evidence of mutational and selective pressure for loss of p16ink4 and for microsatellite instability. *J Invest Dermatol* **114**: 14–20

Ruiz A, Puig S, Lynch M, Castel T and Estivill X (1998) Retention of the CDKN2A locus and low frequency of point mutations in primary and metastatic cutaneous malignant melanoma. *Int J Cancer* **76**: 312–316

Schuchter LM (1997) Melanoma and other skin neoplasms. *Curr Opin Oncol* **9**: 175–177

Smith-Sorenson B and Hoving E (1996) CDKN2A [P16(INK4A)] somatic and germline mutations. *Hum Mutat* **7**: 294–303

Soufir N, Avril M-F, Chompret A, Demenais F, Bombled J, Spatz A, Stoppa-Lyonnet D, the French Familial Melanoma Study Group, Bernard J and Bressac-de Paillerets B (1998) Prevalence of p16 and CDK4 germline mutations in 48 melanoma-prone families in France. *Hum Mol Genet* **7**: 209–216

Talwalkar VR, Scheiner M, Hedges LK, Butler MG and Schwartz HS (1998) Microsatellite instability in malignant melanoma. *Cancer Genet Cytogenet* **104**: 111–114

Wagner SN, Wagner C, Briedigkeit L and Goos M (1998) Homozygous deletion of the p16INK4a and the p15INK4b tumour suppressor genes in a subset of human sporadic cutaneous malignant melanoma. *Br J Dermatol* **138**: 13–21

Zuo L, Weger J, Yang Q, Goldstein AM, Tucker MA, Walker GJ, Hayward N and Dracopoli NC (1996) Germline mutations in the $p16^{INK4a}$ binding domain of CDK4 in familial melanoma. *Nature Gen* **12**: 97–99

 *© 2000 Cancer Research Campaign*
